# Polymeric Sustained-Release Chlorhexidine Coating on Gutta-Percha Points for Prolonged Intracanal Antimicrobial Delivery: An In Vitro Study

**DOI:** 10.3390/pharmaceutics18040405

**Published:** 2026-03-25

**Authors:** Yarden Sabah, Nathanyel Sebbane, Michael Friedman, Irith Gati, Itzhak Abramovitz, Nurit Kot-Limon, Doron Steinberg

**Affiliations:** 1 Biofilm Research Laboratory, The Institute of Biomedical and Oral Research (IBOR), The Faculty of Dental Medicine, The Hebrew University of Jerusalem, Jerusalem 9112102, Israel; yarden.sabah@mail.huji.ac.il (Y.S.); nathanye.sebbane@mail.huji.ac.il (N.S.); dorons@ekmd.huji.ac.il (D.S.); 2 Department of Endodontics, The Faculty of Dental Medicine, The Hebrew University-Hadassah, Jerusalem 9112102, Israel; nurit.kot@mail.huji.ac.il; 3 “Bina” Program, Faculty of Dental Medicine, The Hebrew University-Hadassah, Jerusalem 9112102, Israel; 4 Institute for Drug Research, Faculty of Medicine, The Hebrew University-Hadassah, Jerusalem 9112102, Israel; michaelf@ekmd.huji.ac.il (M.F.); irith.gati@mail.huji.ac.il (I.G.)

**Keywords:** sustained-release device, chlorhexidine, gutta-percha, endodontic biofilm, *Enterococcus faecalis*, *Candida albicans*, root canal disinfection

## Abstract

**Background**: Persistent endodontic infections involving *Enterococcus faecalis* and *Candida albicans* are a major cause of root canal treatment failure. Although conventional irrigants, such as sodium hypochlorite and chlorhexidine (CHX), exhibit strong immediate antimicrobial activity, microbes may survive and recover from the initial antimicrobial effect, hence limiting their effectiveness, especially in complex root canal anatomies and in the apical terminus of the tooth. Antibacterial dressing techniques were not proven satisfactory due to depletion of the antibacterial component or difficulty in spreading it evenly along the entire root canal. This study aimed to develop and evaluate the antimicrobial efficacy and release characteristics of a novel sustained-release device (SRD), delivering CHX via gutta-percha points coated with a sustained-release formulation used as a temporary intracanal medicament. **Methods**: Gutta-percha points were coated with two sustained-release CHX varnishes (CHX1 and CHX2) or a placebo and assessed in vitro. Antimicrobial activity against *E. faecalis* and *C. albicans* was evaluated using agar diffusion assays over time. Release kinetics were analyzed using Rhodamine-labeled SRD in a 3D-printed acrylic molar tooth model via fluorescence microscopy. Additionally, biofilm-infected acrylic molar teeth were treated with a placebo, a single 2% CHX irrigation, or SRD-coated gutta-percha points placed as an intracanal dressing prior to obturation. Microbial viability was quantified by colony-forming unit (CFU/mL) analysis from root canals and gutta-percha points. Statistical analysis was performed using one-way ANOVA followed by Tukey’s post hoc multiple comparison test (*p* < 0.05). **Results**: SRD-coated gutta-percha points demonstrated sustained antimicrobial activity for up to 21 days against *E. faecalis* and 19 days against *C. albicans*. Fluorescence analysis, in an acrylic tooth model, confirmed continuous release for up to 15 days, with pronounced diffusion in the isthmus and palatal canals. In biofilm-infected acrylic teeth models, SRD treatment resulted in a significant reduction of 2–3 log_10_ CFU/mL compared to placebo groups (*p* < 0.001) and prevented microbial rebound over the 14-day observation period. In contrast, a single application of 2% CHX solution showed only transient reduction followed by regrowth. **Conclusions**: Sustained-release CHX delivery via polymer-coated gutta-percha points provided prolonged antimicrobial activity against bacterial and fungal biofilms compared to conventional single-dose CHX application in this in vitro model. These findings support the potential use of coated gutta-percha points as a removable intracanal drug delivery platform prior to final obturation, although further studies incorporating direct-release quantification and in vivo validation are required before clinical translation.

## 1. Introduction

The role of bacteria in the etiology of endodontic disease was established by Kakehashi et al. [1], who demonstrated that germ-free rats responded with spontaneous healing to pulp surgical exposures while conventional rats that underwent pulp exposure developed pulp necrosis and periradicular lesions due to oral bacteria. Since then, endodontic treatment has focused on eliminating or minimizing microbial presence in the root canal system. The traditional approach involves a combination of chemo-mechanical procedures, including irrigation with agents such as sodium hypochlorite (NaOCl), chlorhexidine (CHX), and iodine-potassium iodide (IKI), to enhance both mechanical debridement and disinfection [2]. However, despite these efforts, the complex anatomy of root canals often provides a safe ecological niche for microbes, therefore contributing to treatment failures [3]. While endodontic infections have traditionally been regarded as primarily bacterial in origin, there is increasing evidence that non-bacterial microorganisms, particularly fungal species such as *Candida albicans*, may also contribute to persistent and therapy-resistant endodontic infections, highlighting a broader microbial challenge in root canal disinfection.

Root canal dressing with antibacterial material has been suggested as an additional approach to control intracanal infection prior to root canal obturation, however, its therapeutic effect is controversial [4]. Furthermore, Chavez De Paz et al. [5] have shown that under clinical conditions, infected, rinsed, and dressed root canals were not rendered bacteria-free until the fourth session of root canal dressing. Keeping in mind that root canals are mostly dressed once or twice for a week or two may explain the futility of the present dressing techniques and calls for a more predictable approach to infection control.

*Enterococcus faecalis*, a Gram-positive bacterium, is particularly notorious for its role in failed root canal treatments. Its resilience, ability to penetrate dentinal tubules, and capacity to form biofilms make it a formidable challenge in the treatment of endodontic infections [6]. Studies have shown that *E. faecalis* is present in 24% to 77% of failed endodontic treatments and is often the predominant organism in these cases [7,8,9]. Additionally, the presence of *Candida* species, particularly *Candida albicans*, has been noted in persistent root canal infections. *C. albicans* has been associated with endodontic failures due to its ability to form resistant biofilms and withstand common intracanal disinfectants, further complicating treatment outcomes [10]. A broader analysis by Waltimo et al. [11] found that yeasts, particularly *C. albicans*, were not only present in cases of apical periodontitis but may also play a significant role in the chronicity of such infections, highlighting their underestimated importance in endodontic pathology.

To address these persistent infections, antimicrobial intracanal medicaments such as calcium hydroxide, CHX, and various bioceramic materials have been employed to supplement mechanical debridement. However, these medicaments often fall short of providing long-term disinfection, particularly in the face of resilient pathogens, like *E. faecalis* and *C. albicans* [12,13]. One of the key challenges with these medicaments is their rapid depletion, as CHX and other agents are washed out or neutralized by dentinal proteins and biofilm resistance mechanisms, limiting their effectiveness over time [14].

Root canal medication is technique-sensitive and biologically challenging. The quality of the antibacterial effect of medication depends on the number of medication applications and the quality of the technique [15]. Quality control of medication placement is presently insufficient, resulting in a potential ineffective under-dose medication of the root canal, especially in complex anatomies and the apical end of the root canal, which results in an inability to produce a positive clinical effect [16].

The advancements in the technology of sustained-release drug delivery systems offer a promising alternative to traditional intracanal medicaments. Sustained-release technology allows for the gradual and controlled release of therapeutic agents over an extended period, ensuring that the active substance remains at therapeutic levels along the root canal. This technology offers multiple benefits, including prolonged antimicrobial activity, reduced need for repeated applications, and improved patient compliance [17,18].

Research has demonstrated that sustained-release systems can effectively deliver antimicrobial agents, such as CHX, directly into the dentinal tubules, thereby overcoming the limitations of conventional irrigation techniques, which often fail to maintain therapeutic concentrations over time [19]. These delivery systems enable a prolonged release of the active agent, allowing continuous antimicrobial activity within the complex anatomy of the root canal system. Recent studies have highlighted the potential of sustained-release formulations—such as CHX-loaded varnishes, hydrogels, and nanoparticles—to improve disinfection efficacy and reduce microbial recolonization between treatment sessions [20].

Earlier attempts to incorporate antimicrobial agents directly into gutta-percha points have also been reported. For example, Oztan et al. [21] evaluated gutta-percha points containing root canal medicaments and demonstrated antimicrobial activity against *Enterococcus faecalis* and yeasts in vitro. However, the experimental model used in that study involved bovine anterior teeth with relatively simple canal anatomy and did not include the apical portion of the root canal system. In addition, antimicrobial activity was primarily evaluated as a short-term dressing effect without longitudinal monitoring of microbial regrowth. These limitations highlight the need for models that better simulate the anatomical complexity of clinical root canal systems and allow evaluation of sustained antimicrobial effects over time.

In endodontics, sustained-release drug delivery systems have been explored in various forms, such as nanoparticles, and other polymer-based devices. These systems offer the potential to enhance the substantivity of the active agent, a pharmacological critical factor in maintaining long-term antimicrobial activity [22]. CHX is known for its substantivity, allowing it to adhere to tissue surfaces and continue exerting its antimicrobial effects even after the initial application [23]. CHX’s ability to bind to hydroxyapatite and slowly release over time ensures a sustained antimicrobial presence in the root canal, offering extended protection against bacterial colonization [24,25]. Its cationic biocide nature enables it to breach microbial cell walls effectively, leading to the destruction of a wide array of bacteria and fungi—a critical step in curbing infections within the root canal [26,27]. These attributes underscore CHX’s unique role in the comprehensive disinfection of the root canal, which is essential for the successful treatment of pulp and periradicular diseases, and sets CHX apart from other disinfectants that may dissipate more quickly, leaving the canal vulnerable to reinfection [27]. However, its effectiveness diminishes within days due to washout and neutralization by dentin and proteins, which underscores the need for sustained-release formulations to extend CHX’s therapeutic window [23].

An in vitro study by Heling et al. [28] demonstrated the potential of sustained-release technology in endodontics by evaluating a CHX-loaded SRD for its ability to reduce bacterial populations and prevent secondary infections. The study showed promising results, with the SRD significantly reducing microbial presence over time, highlighting its potential to overcome the limitations of traditional medicaments. Similarly, sustained-release technology has been employed successfully in other areas of dentistry, such as periodontics and the prevention of caries, where it has demonstrated enhanced therapeutic efficacy through controlled, prolonged drug delivery [29].

Building upon earlier approaches using medicated gutta-percha points, the present study applies a sustained-release formulation directly onto conventional gutta-percha points used as a temporary intracanal medicament, thereby transforming a familiar endodontic material into a removable intracanal drug delivery carrier rather than a definitive obturation material. In the present protocol, the coated gutta-percha points were intended to function as an antimicrobial dressing during the inter-appointment period and to be removed prior to final obturation with conventional gutta-percha. Furthermore, this work incorporates longitudinal diffusion mapping in a multi-root acrylic molar model, including an isthmus configuration, combined with time-dependent biofilm suppression analysis for both bacterial and fungal species. This integration of sustained delivery into a temporary intracanal medicament strategy, together with dynamic microbial monitoring, represents a potential translational application of sustained-release technology in endodontic disinfection.

The application of sustained-release technology to combat *E. faecalis* and *C. albicans* in root canal infections is particularly relevant, as these pathogens are adept at resisting conventional treatment methods. By delivering CHX in a sustained-release format, it is possible to maintain high concentrations of the antimicrobial agent within the root canal for extended periods, preventing bacterial/fungal recovery and recolonization and potentially improving overall treatment outcomes.

This approach is intended to function as a temporary intracanal dressing during the inter-appointment period, rather than as a definitive obturation material, and should be considered as a proof-of-concept requiring further validation under clinical conditions.

The aim of the present study is to develop and investigate the efficacy of a novel sustained-release technology for delivering CHX in the root canal system. By coating gutta-percha points with a sustained-release varnish containing CHX, this study will assess the ability of this system to inhibit microbial recovery, particularly of *E. faecalis* and *C. albicans*, thereby improving root canal infection control prior to obturation.

## 2. Materials and Methods

### 2.1. Material

Chlorhexidine di-gluconate (20%; CHX) was purchased from Sigma Company (St. Louis, MO, USA).

*Enterococcus faecalis* ATCC 29,212 bacteria were obtained from a −80 °C stock of the Biofilm Research laboratory (Institute of Biomedical and Oral Research (IBOR), The Hebrew University of Jerusalem, Jerusalem, Israel). The initial bacterial culture was prepared by incubating 100 μL of the bacteria stock in 10 mL of brain heart infusion broth (BHI; Acumedia, Lansing, MI, USA) for 16–18 h at 37 °C in a combined atmosphere of 95% air and 5% CO_2_.

*Candida albicans* SC5314 were obtained from a −80 °C stock from the Biofilm Research laboratory. They were grown for 24–48 h at 30 °C on potato dextrose agar (PDA; Neogen, Lansing, MI, USA) plates. The yeast cells were resuspended at an OD_600_ = 0.05 in RPMI medium (Sigma-Aldrich, St. Louis, MO, USA) and used for biofilm assays by incubation at 37 °C.

The SRDs were prepared similarly to the method [30].

CHX1 varnish: 300 mg chlorhexidine (Sigma-Aldrich, St. Louis, MO, USA), 600 mg ethylcellulose (Sigma-Aldrich, St. Louis, MO, USA), 60 mg PEG400 (Sigma-Aldrich, St. Louis, MO, USA), 600 mg hydroxypropylcellulose (Sigma-Aldrich, St. Louis, MO, USA), and 10 mL ethanol.

CHX2 varnish: 1.5 g chlorhexidine diacetate (Sigma-Aldrich, St. Louis, MO, USA), 3 g ethylcellulose (Ethocel^®^N-100, Hercules Inc., Wilmington, DE, USA; Ashland, Wilmington, DE, USA), 0.3 g polyethylene glycol (PEG-400, Sigma-Aldrich, St. Louis, MO, USA), and 50 mL ethanol.

Placebo varnish: 3 g ethylcellulose (Ethocel^®^N-100, Hercules Inc., USA; Ashland, USA), 0.3 g polyethylene glycol (PEG-400, Sigma-Aldrich, St. Louis, MO, USA), and 50 mL ethanol.

Gutta-percha points (Dentsply, Sirona, Charlotte, NC, USA) were coated by the dipping method. The points were immersed in the varnish solutions (3-time dipping) and then air-dried under sterile conditions for 24 h. The coated gutta-percha points were stored in sterile containers until the beginning of the experiment. The average net dry weight of the SRD was 0.001 g, as measured by weighing the gutta-percha points before and after coating [31].

### 2.2. Effect of the Sustained-Release Technology on E. faecalis and C. albicans Growth

The antibacterial effects of CHX on *E. faecalis* and *C. albicans* were evaluated through two separate experiments. Both experiments utilized sustained-release technology by coating gutta-percha points with CHX varnishes of varying concentrations.

The antibacterial and antifungal activity of these coated gutta-percha points was tested using an agar diffusion method. Separate sterile agar plates were inoculated with bacterial suspensions of *E. faecalis* or fungal suspensions of *C. albicans*. Coated gutta-percha points, including those coated with CHX1, CHX2, and the placebo varnish, were placed onto the inoculated agar plates and incubated at 37 °C for 24 h. Each day, the gutta-percha points were transferred to fresh agar plates seeded with new bacterial or fungal suspensions. The transfer process was repeated daily for 21 days. The antimicrobial efficacy was assessed by measuring the zone of inhibition—where bacterial or fungal growth was inhibited—using a standardized imaging and analysis protocol. Plates were photographed using a fixed camera setup, standardized illumination conditions, and identical exposure settings. Images were converted to grayscale in ImageJ (NIH, Bethesda, MD, USA, version 1.54g), and inhibition zones were measured by manually outlining the halo perimeter using the calibrated scale (90 mm Petri dish diameter). Measurements were performed independently by two blinded examiners, and the mean value was used for analysis. The agar diffusion assay was performed with *n* = 4 per group, consistent with established microbiological diffusion testing standards. Although the per-group replication was modest, daily longitudinal transfer over 21 days generated repeated biological measurements that strengthened internal reliability.

This method allowed for an evaluation of both the extent and the duration of the antimicrobial effect of each formulation. The sustained-release capability of each varnish was analyzed based on the changes in the inhibition halo over time, providing data to determine if higher concentrations of CHX (as in CHX2) offered greater antimicrobial benefits or if they reached an efficacy plateau [6,32].

### 2.3. Fluorescent Model in Acrylic Tooth for Assessing Sustained-Release Technology in Root Canal Treatments

The Rhodamine-labeled SRD was prepared similarly to the CHX1/CHX2 formulations described above, with the addition of 25 mg of Rhodamine B, and the mixture was heated for 15 min to ensure full dissolution before being stored at room temperature.

Gutta-percha points (Dentsply, Sirona, USA) were coated with the Rhodamine SRD formulation. A green gutta-percha point was designated for the palatal canal, while two blue gutta-percha points were used per tooth for the mesio-buccal canals (green gutta-percha point size 35/0.04 and blue gutta-percha points’ size 30/0.04; Dentsply, Sirona USA). The gutta-percha points were coated using the same dipping method described above.

The experimental setup utilized transparent acrylic tooth models (FKG 3D tooth #26, ref 999.70.07A.xx, FKG Dentaire, La Chaux-de-Fonds, Switzerland) representing multi-rooted upper permanent molars. The disto-buccal root of each model was removed using a dental low-speed handpiece with a disk attachment, allowing for an unobstructed view of the mesio-buccal root from a proximal angle. Red wax in the pulp chamber was removed mechanically using an excavator. The mesio-buccal and palatal canals were prepared using ProTaper Next™ Ni-Ti files (Dentsply Maillefer, Baillaigues, Switzerland), with the mesio-buccal canals instrumented up to X2 (25/0.06) and the palatal canal prepared up to X3 (30/0.07), followed by saline and ethanol irrigation. The mesio-buccal root model included two distinct canals connected by an isthmus, reflecting the common anatomical configuration of maxillary molars. The teeth were then soaked in ethanol for 24 h to remove any residual wax from the canals, washed extensively in sterile PBS, and then air-dried under sterile conditions for 24 h.

Model setup: Following root canal preparation, the coated gutta-percha points were inserted into the canals. A coronal seal was created using Coltosol, and the samples were wrapped in Parafilm. An apical seal was established using 1% agar dissolved in distilled deionized water (DDW). The prepared teeth were placed in 24-well plates and incubated at 37 °C under humid conditions. The control groups consisted of teeth with saline and either uncoated gutta-percha or placebo-coated gutta-percha points.

Fluorescent analysis: A jig was designed to ensure consistent positioning and repeatable measurements. An impression of the tooth crown was taken using polysiloxane precision impression material (Optosil, Kulzer GmbH, Hanau, Germany) and placed inside a 5 cm × 2 cm plastic cube positioned on a microscope plate for standardized imaging. Fluorescent images were captured using a Nikon SMZ25 stereomicroscope (Nikon, Tokyo, Japan) with excitation and emission filters set at 560 nm and 630 nm, respectively. Fluorescence intensity was analyzed using NIS-Elements software (Nikon, Tokyo, Japan; available at: https://www.microscope.healthcare.nikon.com/products/software/nis-elements, accessed on 22 March 2026). Four areas of interest were delineated: one area for the palatal canal, two for the mesio-buccal canals, and one for the isthmus between the two mesio-buccal canals. These setups were consistent across all samples. Fluorescent pixels within the defined regions were quantified, with the fluorescence threshold set above the autofluorescence level of the control teeth. The fluorescence intensity per area was then recorded and analyzed to assess the diffusion of the sustained-release formulation [33].

### 2.4. Effect of Sustained-Release Technology on Biofilms Formed by E. faecalis and C. albicans in Infected Acrylic Tooth Models

Experimental setup: This experiment evaluated the efficacy of gutta-percha points coated with sustained release of CHX in reducing bacterial and fungal biofilms in acrylic tooth root canals. Twelve transparent acrylic tooth models (FKG 3D tooth #26) were used to simulate human molar root canals for each pathogen, sterilized by immersion in ethanol for 24 h and subsequently air-dried to prevent ethanol interference with bacterial or fungal viability.

Biofilm formation: The sterilized teeth were incubated with *C. albicans* or *E. faecalis* for one week to promote biofilm formation in the root canals. During this period, the culture media were refreshed by replacing two-thirds of the medium every two days [34]. The culture was checked daily for contamination.

Treatment groups: Following biofilm formation, the teeth were divided into three groups: a placebo group treatment (coated gutta-percha points without CHX), a group treated with CHX as a single application (one-minute irrigation with 2% CHX), and a group treated with gutta-percha points coated with a sustained-release CHX varnish.

Sampling strategy: The treatment groups were sampled to evaluate the prolonged dynamic antimicrobial activity of the formulations. Initial sampling was conducted 24 h post-treatment, followed by additional samples collected at 3, 8, and 14 days post-treatment. This sampling schedule was selected to monitor both immediate and sustained effects of the CHX treatment.

Sample collection and processing: At each sampling point, teeth were subjected to a 5 min sonication in a water bath to dislodge the biofilm from the tooth surfaces. Sterile paper points (Pearl Dent Co., Ltd., Ho Chi Minh City, Vietnam) were inserted into each of the three canals per tooth for 30 s to absorb canal contents, including biofilm constituents. Four paper points were collected per tooth, due to the splitting of one canal into two branches. These paper points were immediately transferred into sterile Eppendorf tubes containing 1 mL of RPMI (for *C. albicans*) or BHI (for *E. faecalis*). The suspension was vortexed for 30 s to elute the fungi or bacteria from the paper points. Serial dilutions (1:10, 1:100, etc.) of the suspension were prepared, and aliquots were plated on PDA plates (for *C. albicans*) or BHI plates (for *E. faecalis*) to quantify colony-forming units (CFUs).

Incubation and CFU quantification: The plated aliquots were incubated in 95% air/5% CO_2_ (*v*/*v*) at 37 °C for 24 h, after which the colonies were enumerated. From each tooth, eight plates were prepared, allowing comprehensive quantification of the fungal or bacterial load. The results were used to calculate the CFU/mL in the original samples by applying the following formula: CFU/mL = (number of colonies × dilution factor)/volume plated. The CFU values were analyzed statistically to compare the effectiveness of the placebo, single CHX treatment, and sustained-release CHX gutta-percha points groups.

Gutta-percha point residual microbial activity: In parallel, the gutta-percha points used in the sustained-release group were also checked for residual microbial activity. Each gutta-percha point was placed in an Eppendorf tube containing 1 mL of RPMI or BHI, and the number of bacteria/fungi was enumerated, as described above. The CFU counts from the gutta-percha points were compared to the biofilm load within the tooth canals to assess the ongoing antimicrobial effect of the sustained-release technology.

Image analysis: ImageJ software was used to capture and analyze bacterial and fungal growth and CFU quantification [34].

Each tooth was considered one independent experimental unit (*n* = 8 per group). CFU values obtained from multiple canals within the same tooth were averaged prior to statistical analysis to avoid pseudo-replication.

### 2.5. Statistical Analysis

The results are presented as mean ± standard deviation (SD). Statistical analyses were performed using one-way analysis of variance (ANOVA) followed by Tukey’s post hoc multiple comparison test for intergroup comparisons. Statistical significance was defined as *p* < 0.05. Adjustments for multiple comparisons were applied using Tukey’s post hoc test to control for type I error. For biofilm experiments involving repeated sampling over time, treatment and time were considered independent factors. Multiple comparisons were corrected using Tukey’s post hoc test. Statistical significance was set at *p* < 0.05.

Due to the exploratory design, a full two-way ANOVA model was not applied.

No formal priori power analysis was performed, as this investigation was designed as an exploratory in vitro proof-of-concept study.

## 3. Results

### 3.1. Coated Gutta Percha Points with the Sustained-Release Technology Reduced E. faecalis and C. albicans Growth for 21 Days and 19 Days, Respectively

In our initial investigation, we coated gutta-percha points with the different varnishes, CHX1 and CHX2, and placed them on agar plates pre-seeded with *E. faecalis* or *C. albicans*. The gutta-percha points were transferred daily to a new plate, while we measured the growth inhibition halo in cm^2^.

**Figure 1:**
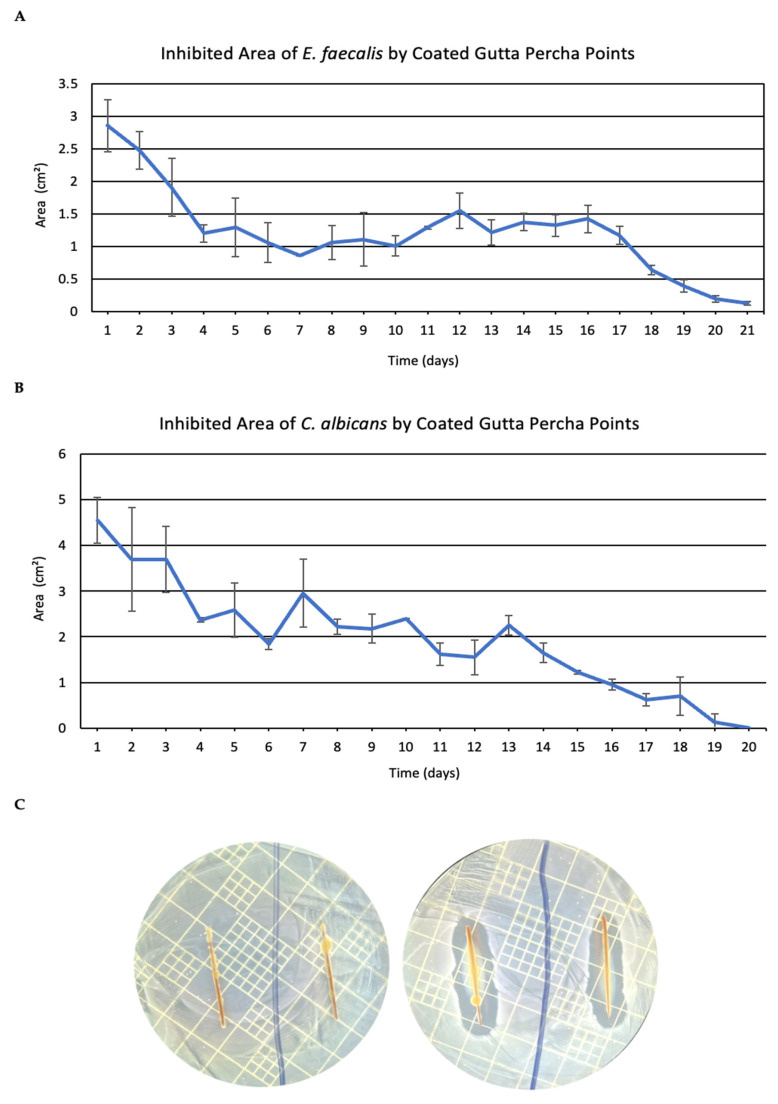
The effect of coated gutta-percha points with sustained-release technology on plate growth. Halo of delay measured around the gutta-percha points. (**A**) Inhibition area and killing of *E. faecalis* that was exposed to the sustained-release technology gutta percha points. (**B**) Inhibition area and killing of *C. albicans* that was exposed to the sustained-release technology on synthetic gutta-percha points. *n* = 4. (**C**) Representative agar diffusion images at Day 7 comparing placebo-coated (left) and CHX2-coated (right) gutta-percha points against *E. faecalis*. Scale reference: Petri dish diameter = 90 mm. *n* = 4. Statistical significance was determined using one-way ANOVA followed by Tukey’s post hoc test.

It can clearly be seen (Figure 1) that the varnishes provide an effective release of the antibacterial composition that prevented bacterial growth for about three weeks (with a decrease during the third week). The placebo group, composed of coated gutta-percha points with placebo sustained-release technology, did not affect bacterial growth (halo was 0 every day).

Figure 1A shows the average inhibition of two different types of gutta-percha points. The first group (blue) was composed of two gutta-percha points, Taper 04, while the second group (red) was composed of two synthetic gutta-percha points. It can be observed that the varnishes provide an effective release of the antibacterial composition, which killed the bacteria present on the plate, for the two types of coated gutta-percha points. Both formulations exhibit a similar dose-dependent inhibition profile.

The growth inhibition of *C. albicans* by coated gutta-percha points is higher during the first days of the experiment and lasts for 21 days. Both formulations exhibit a similar time-dependent effect (Figure 1B). Representative agar diffusion plates are shown in Figure 1C, visually confirming the persistent inhibition halos observed in the SRD groups compared to placebo controls.

Statistical analysis using one-way ANOVA followed by Tukey’s post hoc test demonstrated a significant effect of treatment on the inhibition zone area over time (*p* < 0.001). Post hoc comparisons revealed that both CHX1 and CHX2 groups exhibited significantly larger inhibition zones compared to the placebo from Day 1 to Day 19 (*p* < 0.05), while no significant difference was observed between CHX1 and CHX2 (*p* > 0.05).

Since CHX1 and CHX2 demonstrated similar inhibition kinetics without statistically significant differences, their values were averaged for graphical presentation. The placebo group showed no measurable inhibition (0 cm^2^) at all time points.

### 3.2. Sustained Rhodamine Release Demonstrates Prolonged Diffusion in Complex Root Canal Anatomy

**Figure 2:**
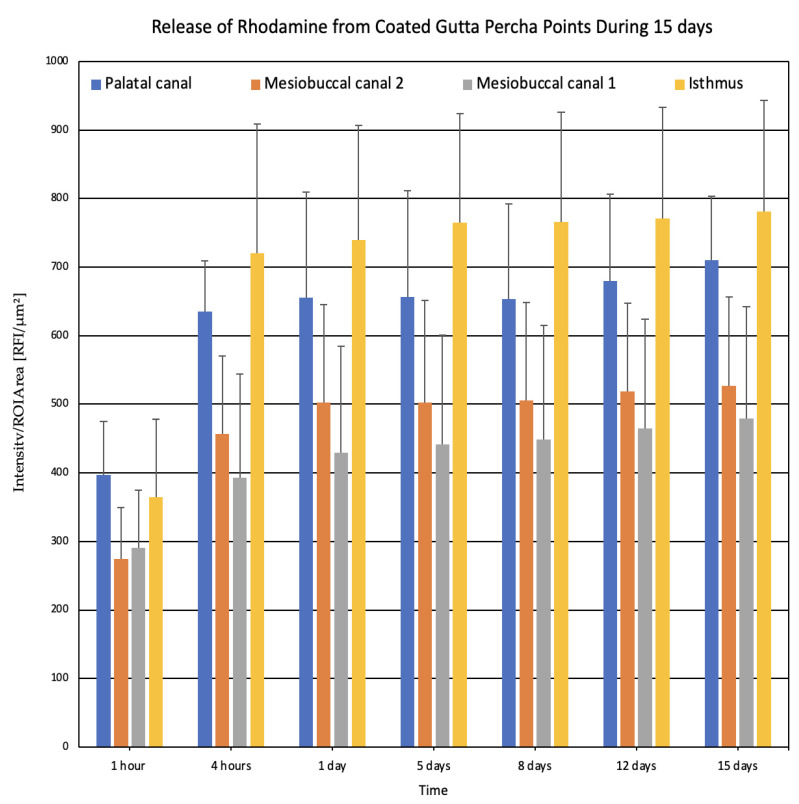
The intensity of Rhodamine fluorescence in four distinct anatomical areas: palatal canal—dark blue, mesio-buccal canal 1—green, mesio-buccal canal 2—orange, and the isthmus between the mesial canals—light blue. Fluorescence intensity was measured over a 15-day period to assess the release kinetics of the SRD. *n* = 7.

Figure 2 illustrates the release kinetics of Rhodamine from the SRD applied to gutta-percha points over a 15-day period, as assessed by fluorescence intensity in different regions of the acrylic tooth model. Over time, the fluorescence intensity was highest in the isthmus region, followed by Canal P, and the two mesio-buccal canals exhibited the lowest fluorescence values. From 1 h to 1 day post-application, the fluorescence intensity increased across all anatomical tooth regions, with the isthmus showing slightly elevated values compared to the canals. This suggests an initial uniform release of Rhodamine from the SRD. However, as time progressed, differences became more pronounced. By Day 5, the isthmus showed significantly higher fluorescence intensity (approximately 780 RFU/μm^2^), indicating a sustained and higher concentration of the Rhodamine release in this region. The palatal canal also maintained a higher fluorescence level (around 650 RFU/μm^2^) compared to the mesial canals, which exhibited lower levels (400–500 RFU/μm^2^). This trend persisted over a 15-day period, and the progression from 1 h to Day 15 reached about ×1.5–2 in the different anatomical areas. The isthmus consistently showed the highest Rhodamine concentration.

We can observe a slight but constant increase in fluorescence throughout the experiment; until Day 15, the overall fluorescence values remained substantial, confirming that the sustained-release system provides a prolonged delivery. These results support the efficacy of the SRD in maintaining Rhodamine presence in critical areas of the root canal system, particularly in the isthmus, which is often challenging to disinfect with conventional methods.

Rhodamine was incorporated as a fluorescent tracer to visualize spatial diffusion behavior within the acrylic root canal model. While Rhodamine fluorescence does not represent a direct quantitative measurement of CHX concentration, it provides qualitative confirmation of sustained diffusion from the coated gutta-percha into the complex canal anatomies over time.

### 3.3. The Sustained-Release Technology Significantly Decreased Biofilms Formed by E. faecalis in Infected Acrylic Tooth Models

**Figure 3:**
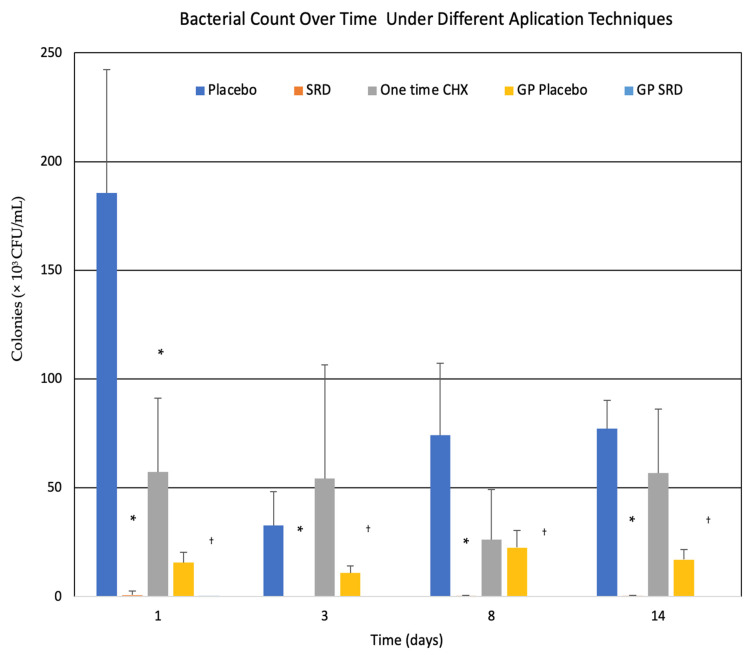
Bacterial count over time in *E. faecalis* biofilm-infected acrylic tooth models treated with different antimicrobial approaches. CFUs measured in × 10^3^ CFU/mL over a 14-day period across various treatment groups. Bacterial count over time: average of CFU in teeth canals, depending on the treatment throughout the 14 days. Bars represent the mean CFU values obtained from the canals of each tooth, depending on the treatment group. Treatment groups are represented by the following colors: blue (placebo): canals treated with placebo sustained-release device (SRD) coating without chlorhexidine (CHX); orange (SRD): canals treated with the sustained-release CHX coating applied directly in the canal system; gray (one-time CHX solution): canals treated with a single application of 2% CHX solution; yellow (GP-placebo): gutta-percha points coated with placebo SRD (without CHX) inserted into the canals; light blue (GP SRD): gutta-percha points coated with CHX-containing SRD inserted into the canals. * *p* < 0.001 compared to placebo group at the corresponding time point (SRD vs. placebo and one-time CHX vs. placebo). ^†^ *p* < 0.001 compared to GP-placebo group (GP-SRD vs. GP-placebo). *n* = 8. Statistical significance was determined using one-way ANOVA followed by Tukey’s post hoc test.

The series of graphs provided in Figure 3 demonstrate the bacterial count over time in different treatment groups, focusing on *E. faecalis* biofilm formation in acrylic tooth models. The primary outcome assessed the bacterial colony-forming units (CFUs), expressed as ×10^3^ CFU/mL, over a 14-day period across several groups: placebo (gutta-percha points coated without CHX), single application of CHX solution, and the SRD coating on gutta-percha points.

In the placebo group (Figure 3), bacterial counts in canals remained consistently high, with no significant reduction in CFU/mL over time. At Day 1, the bacterial load exceeded 180 × 10^3^ CFU/mL, decreased at Day 3, and from Day 8 remained above 50 × 10^3^ CFU/mL until Day 14, indicating that no effective antimicrobial activity was demonstrated in the presence of placebo SRD. The placebo-coated gutta-percha points (Figure 3) also exhibited similar trends, with high bacterial counts maintained in all canals, confirming that the coating without active agents did not mitigate *E. faecalis* growth without exception due to anatomical area.

In contrast, the single-application CHX solution group (Figure 3) showed an initial reduction in bacterial counts at Day 1, where CFU/mL dropped to approximately 50 × 10^3^ CFU/mL, but bacterial growth rebounded at Days 3 and 14, with a slight decrease at Day 8. This result suggests that while CHX is effective in the short term, its antibacterial effect diminishes over time, allowing biofilm recovery and bacterial recolonization.

The sustained release of CHX from the SRD dramatically reduced bacterial CFUs to less than 3 × 10^3^ CFU/mL by Day 1 and maintained low bacterial counts in all canals from Day 3 (<2 × 10^3^ CFU/mL) throughout the 14-day period (Figure 3). The near-complete suppression of *E. faecalis* biofilm formation indicates that the SRD was highly effective in maintaining prolonged antimicrobial activity, preventing bacterial rebound even after two weeks. This sustained bacterial suppression was consistently observed across the palatal, mesio-buccal, and distal canals, with minimal CFUs detected on the gutta-percha points throughout the duration of the experiment (Figure 3). In addition, almost no bacteria were found on the SRD-coated gutta-percha compared to placebo-coated.

### 3.4. The Sustained-Release Technology Significantly Decreased Biofilms Formed by C. albicans in Infected Acrylic Tooth Models

**Figure 4:**
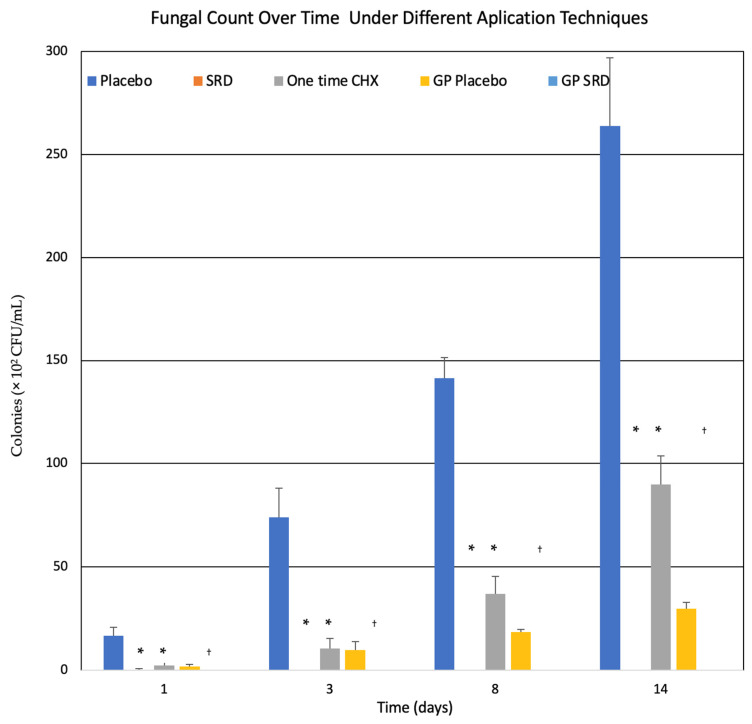
Fungal count over time in *C. albicans* biofilm-infected acrylic tooth models treated with different antifungal approaches. CFUs measured in ×10^3^ CFU/mL over a 14-day period across various treatment groups. Fungal count over time: average of CFU in teeth canals, depending on the treatment throughout the 14 days. Bars represent the mean CFU values obtained from the canals of each tooth, depending on the treatment group. Treatment groups are represented by the following colors: blue (placebo): canals treated with placebo sustained-release device (SRD) coating without chlorhexidine (CHX); orange (SRD): canals treated with the sustained-release CHX coating applied directly in the canal system; gray (one-time CHX solution): canals treated with a single application of 2% CHX solution; yellow (GP-placebo): gutta-percha points coated with placebo SRD (without CHX) inserted into the canals; light blue (GP SRD): gutta-percha points coated with CHX-containing SRD inserted into the canals. * *p* < 0.001 compared to placebo group at the corresponding time point (SRD vs. placebo and one-time CHX vs. placebo). ^†^ *p* < 0.001 compared to GP-placebo group (GP-SRD vs. GP-placebo). *n* = 8. Statistical significance was determined using one-way ANOVA followed by Tukey’s post hoc test.

The graphs displayed in Figure 4 illustrate the progression of fungal CFUs over time, focusing on *C. albicans* biofilm growth within acrylic tooth models. The study assessed CFU counts expressed as ×10^2^ CFU/mL across a 14-day period, comparing the effects of different treatments: placebo-SRD, a single application of CHX, and CHX-SRD coating applied to gutta-percha points.

In the SRD placebo group (Figure 4), there was a substantial increase in fungal counts as time progressed. While fungal levels remained relatively low at Day 1, the growth accelerated after Day 3, surpassing 70 × 10^2^ CFU/mL, and reaching over 250 × 10^2^ CFU/mL by Day 14. This result confirms that without an active antifungal agent, *C. albicans* colonization may rapidly develop over time. The placebo-coated gutta-percha points (Figure 4) presented similar results, with fungal populations remaining high across all tooth canals, including the palatal, mesio-buccal, and distal regions.

The single application of the CHX solution group (Figure 4) initially showed a more promising response, with fungal counts suppressed below 20 × 10^2^ CFU/mL at Day 1. However, this effect diminished after Day 3, as fungal counts steadily increased, reaching over 100 × 10^2^ CFU/mL by Day 14. This suggests that although CHX provides a temporary reduction in fungal biofilms, its antifungal action is not sustained, allowing *C. albicans* to proliferate once the immediate effect wears off.

In contrast, the most effective fungal suppression was observed in the CHX-SRD group (Figure 4). By utilizing the sustained-release technology, fungal CFUs were reduced to less than 1 × 10^2^ CFU/mL by Day 1, and the antifungal effect remained consistent through Day 14. Notably, fungal growth was virtually eliminated in all canals (palatal, mesio-buccal, and distal), as evidenced by the low CFU values in the CHX-SRD-coated gutta-percha points group (Figure 4). The prolonged antifungal effect provided by the SRD ensures continuous inhibition of *C. albicans* biofilm formation, preventing recolonization and demonstrating the long-term effectiveness of this delivery system. In addition, almost no fungi were found on the SRD-coated gutta-percha compared to placebo-coated.

Compared to the placebo and single-application CHX groups, the SRD system exhibited prolonged antifungal efficacy within this in vitro model, providing sustained antifungal activity over a two-week period. The gradual release of CHX from the SRD was key to maintaining constant antifungal pressure, which is particularly important in preventing fungal regrowth in complex root canal systems. These findings underscore the importance of sustained antifungal presence for ensuring comprehensive infection control, particularly in anatomically challenging regions like the isthmus and distal canals, where traditional treatments often struggle to reach and eliminate fungal biofilms.

## 4. Discussion

This study demonstrates the antimicrobial efficacy of a novel sustained-release CHX coating applied on gutta-percha points in simulated root canal systems, showing significant and prolonged intracanal suppression of both *E. faecalis* and *C. albicans* biofilms. These findings support the concept of sustained-release CHX as a potential adjunctive strategy in a proof-of-concept in vitro model that may help address certain limitations of conventional disinfection protocols in endodontics. By coating gutta-percha points with a sustained-release formulation, the disinfection phase is extended beyond treatment visits, offering active protection within the limitations of the in vitro experimental setting, simulating the inter-appointment period [3]. These results are within the constraints of the in vitro models applied and require further ex vivo and in vivo validations.

*E. faecalis* is a resilient pathogen frequently associated with secondary endodontic infections and treatment failures due to its ability to invade dentinal tubules and survive periods of nutritional deprivation [6,35]. In this study, the sustained-release CHX coating of gutta-percha points not only rapidly reduced CFU counts but also maintained its antibacterial effect for up to 14 days, contrasting sharply with the transient effects observed in the single application of CHX. This is consistent with findings by Lin et al. [36], who reported that CHX delivered via a slow-release system achieved deeper and more effective disinfection of infected dentin compared to solution-based CHX.

Fluorescent imaging of the Rhodamine-labeled SRD confirmed that drug diffusion reached all anatomical regions of the root canal system, with particularly high retention observed in the isthmus. This region is frequently inadequately disinfected by conventional endodontic protocols due to its narrow, complex anatomy, which limits the penetration of both irrigating solutions and mechanical instruments. Importantly, aggressive instrumentation of isthmus-containing areas is not only ineffective but may be contraindicated, as these regions are often characterized by thin dentinal walls. Excessive mechanical preparation in such anatomies increases the risk of dentin removal, strip perforation, and long-term structural weakening of the tooth, potentially predisposing it to vertical root fracture.

In contrast, the sustained-release delivery system enabled continuous diffusion of the antimicrobial agent into the isthmus without reliance on fluid dynamics, pressure, or extensive mechanical enlargement. This observation highlights a key advantage of sustained-release systems in endodontics: the ability to target hard-to-access anatomical complexities while preserving dentin integrity. However, these advantages remain experimental and should be interpreted cautiously, as they have not yet been validated under clinical conditions.

Rhodamine-SRD, placed in the root canals of the acrylic tooth, was used solely as a qualitative tracer to visualize the spatial diffusion pattern of the tested formulation. No calibration curve was generated, and no quantitative correlation between the fluorescence intensity of Rhodamine and the actual CHX concentration was established. Therefore, fluorescence intensity values should not be interpreted as representing absolute or relative CHX concentrations, but as a demonstration of the release and diffusion capabilities of the SRD technology within the root canal.

While traditional irrigants like NaOCl are effective in rapidly reducing bacterial loads and dissolving organic tissue, their lack of substantivity results in low antimicrobial effects post-irrigation. CHX, in contrast, exhibits substantivity due to its cationic nature, allowing it to bind to dentin and exert prolonged effects [37]. However, even this substantivity is short-term in clinical conditions—lasting a few days at most—due to dentin adsorption and buffering [23]. In addition, this release is not controlled and is subject to the environmental conditions in the ecological niche. Therefore, the concept of embedding CHX directly into an SRD system is particularly attractive, as demonstrated here.

The antimicrobial mechanism of action of CHX has been previously described in our earlier study [38], where it was shown to impact cell membrane integrity, depolarization, and permeability in *E. faecalis*. Propidium iodide staining and membrane potential assays demonstrated that CHX compromises bacterial membrane stability—a mode of action consistent with its known biocidal effect. Morphological evidence from SEM imaging further supported this, showing pore formation and cytoplasmic leakage in CHX-exposed cells—corroborating prior literature that associates CHX with structural disintegration of microbial membranes [39]. These mechanistic findings were not directly evaluated in the present study.

Importantly, CHX showed efficacy not only in reducing planktonic populations but also in disrupting mature biofilms—a key feature of resistant infections. These antibiofilm effects are supported by previous findings, including our earlier work [38], rather than being directly assessed in the present study. Biofilm biomass and EPS production were substantially suppressed by sustained CHX release. These results align with earlier observations that low-concentration CHX can interfere with biofilm formation and structure [40]. Moreover, this suppression extended to virulence factors. Previous gene expression analyses (outside the scope of the current study) have shown that CHX downregulates genes linked to adhesion, cytolysin production, and collagen binding—reducing the pathogenic potential of *E. faecalis* even when not fully eradicated [41].

In the present study, the significant reduction in CFUs in the CHX-SRD groups compared to the placebo and single-application CHX solution groups highlights the prolonged antimicrobial activity of the SRD-coated gutta-percha points compared to the control groups, within the limits of these in vitro models. Rhodamine diffusion data further support this, showing a 1.5–2-fold increase in intensity over 15 days, with the isthmus consistently displaying the highest concentration—likely due to its position between two gutta-percha points. These results emphasize the advantage of SRD in achieving long-term disinfection in anatomically complex regions.

Continuous CHX release maintained antibacterial pressure, preventing recolonization, especially in hard-to-reach areas like the isthmus and distal canals. The MIC and antibiofilm activity values referenced in this study were previously established in our earlier publication [38], where CHX demonstrated significant inhibition of planktonic growth and biofilm formation at low concentrations.

Although sustained antimicrobial pressure prevented regrowth in the present model, prolonged sub-inhibitory exposure could theoretically contribute to adaptive responses or microbial resistance. However, we have chosen CHX as an active agent, as it is an antiseptic material. Its mode of action is mainly non-specific by affecting the integrity of the membrane, and its potential ability of triggering CHX-resistant microbes is very limited. This aspect was not evaluated in the current study, as MIC/MBC values under the experimental conditions were not determined, and should be examined in future investigations using dedicated microbial resistance monitoring and concentration response models.

*C. albicans*—a less frequent, but clinically relevant pathogen in endodontic infections—also showed susceptibility to CHX in the sustained-release device. Although bacterial pathogens like *E. faecalis* are more commonly associated with persistent infections, the presence of *C. albicans* in root canal systems has been linked to endodontic failure due to its ability to form resistant biofilms and survive in harsh conditions [42,43]. These findings support the potential of SRD to target both bacterial and fungal pathogens involved in persistent periapical disease and are in line with previous reports demonstrating the recovery of *C. albicans* from therapy-resistant root canal infections and its reduced susceptibility to conventional chemo-mechanical disinfection.

Similarly to *E. faecalis*, it was found in our study that fungal regrowth was also almost completely prevented across the 14-day period during exposure to CHX-SRD. This is particularly meaningful given that CHX is not traditionally fungicidal in low concentrations; thus, its prolonged availability at sub-cytotoxic levels may be key to controlling fungal biofilms also in vivo. The SRD system demonstrated a clear advantage over both placebo and single-application CHX, maintaining antifungal activity throughout the entire observation period. This sustained antifungal inhibition was observed consistently across all canal types, including the palatal and distal canals, areas that are notoriously difficult to disinfect using conventional methods. The consistent suppression of *C. albicans* suggests that the SRD can provide effective long-term antifungal protection even in anatomically challenging zones where fungal recolonization typically occurs. These findings reinforce the underestimated clinical significance of fungal species in persistent endodontic infections and highlight the potential of SRD-based therapies in targeting both bacterial and fungal biofilms simultaneously. The results presented here mirror those of da Silva et al. [44], who showed that CHX altered *C. albicans* biofilm structure and viability in acrylic systems.

The present study employed mono-species biofilm models of *E. faecalis* or *C. albicans* to allow controlled evaluation of antimicrobial dynamics. However, clinical endodontic infections are polymicrobial and involve complex interspecies interactions. Future studies using multispecies biofilm models and dentin-based systems are necessary to better approximate clinical conditions.

In addition, although biofilm dynamics were assessed over multiple time points, statistical analysis was performed using one-way ANOVA at each time point rather than a two-way ANOVA, integrating both treatment and time factors. Therefore, potential interaction effects between time and treatment were not formally evaluated. Future studies incorporating two-way ANOVA or repeated-measures models would provide a more comprehensive understanding of temporal antimicrobial dynamics.

Studies evaluating sustained-release antimicrobial systems in endodontics have reported variable durations of antibacterial activity. For example, Lee et al. [45] reported antibacterial effects against *E. faecalis* dentinal tubule infection for approximately seven days using a polymer-based CHX delivery system. Similarly, Heling et al. [28] demonstrated dentinal sterilization effects lasting approximately 10 days with a CHX sustained-release device. In contrast, the present system maintained measurable inhibition zones for up to 21 days in agar diffusion assays and suppressed biofilm regrowth for 14 days in the acrylic tooth model. These findings suggest that the sustained-release coating applied directly onto gutta-percha points may provide prolonged antimicrobial activity while integrating the drug delivery function into a conventional obturation component.

Previous studies have also explored medicated gutta-percha points as carriers of antimicrobial agents. Oztan et al. [21] reported that gutta-percha points containing intracanal medications demonstrated antimicrobial effects against *E. faecalis* and yeasts in vitro [21]. However, their experimental model involved bovine anterior teeth with relatively simple canal anatomy and did not include the apical region of the canal system. In addition, antimicrobial evaluation focused primarily on the immediate antimicrobial effect of the medicated points and did not include longitudinal monitoring of microbial regrowth. In contrast, the present study used a multi-root acrylic molar model incorporating complex anatomical features, including an isthmus and apical regions, and evaluated antimicrobial dynamics over time through repeated biofilm monitoring. These methodological differences allow a more comprehensive assessment of sustained antimicrobial activity within anatomically complex root canal environments.

While previous sustained-release systems developed by our group and others have evaluated CHX-containing varnishes and fillers as intracanal medicaments, the present study differs in that the drug delivery system is directly integrated into a conventional obturation material (gutta-percha). The SRD-coated gutta-percha eliminates the need for additional placement steps and transforms a routine filling component into a controlled antimicrobial reservoir. Additionally, the present work incorporates anatomical diffusion mapping within a 3D acrylic molar model and longitudinal biofilm monitoring of both bacterial and fungal species. Because the core material remains conventional gutta-percha, retreatment procedures are expected to follow standard mechanical removal protocols; however, quantitative evaluation of removal efficiency was not performed in the present study.

The release mechanism of the SRD coating is expected to be primarily diffusion-controlled due to the presence of a non-degradable ethylcellulose-based polymeric matrix. In such systems, aqueous penetration into the polymer network creates microscopic diffusion pathways through which the active compound is gradually released. Because the matrix itself does not undergo significant degradation under physiological conditions, drug release is largely governed by passive diffusion rather than polymer breakdown. The initial burst release was not specifically quantified in this study. While antimicrobial activity was sustained over time, early peak concentration dynamics were not analytically characterized. Pharmaceutical kinetic modeling of the release profile of CHX (e.g., Higuchi or Korsmeyer–Peppas models) was not performed in the present study and should be addressed in future pharmaceutical characterization studies.

It is important to emphasize that sustained antimicrobial activity in the present study was inferred from biological assays and qualitative diffusion imaging rather than from direct chemical analytical quantification of chlorhexidine release.

The SRD formulation evaluated in this study is composed of ethylcellulose, hydroxypropyl cellulose, and polyethylene glycol—excipients widely used in pharmaceutical and biomedical applications with established biocompatibility profiles. Chlorhexidine is routinely employed in endodontic irrigation and intracanal medication, as well as in other dental medications, and its cytotoxicity is known to be concentration dependent. The advantage of the sustained-release technology is that the total amount of the drug is low, and the concentrations of the released agent are also low. However, cytotoxicity of the CHX-SRD should be evaluated in future studies.

The potential impact of the coating on the sealing ability of gutta-percha and possible physicochemical interactions between CHX and commonly used endodontic sealers were not evaluated in this study. These parameters are critical for clinical translation and require dedicated investigation. Although the applied coating mass was minimal (~1 mg per point), its potential influence on adaptation, interface integrity, and sealer penetration requires dedicated microleakage and micro-CT investigation.

The clinical translation of this in vitro model must be conducted with caution. The acrylic tooth model was selected to provide standardized anatomical reproducibility and optical accessibility for diffusion assessment and antimicrobial effects in this unique anatomically micro-environment. The use of acrylic teeth eliminates confounding factors, such as dentin buffering, dentinal tubule penetration, smear layer interference, or protein binding—all of which can affect antimicrobial efficacy in natural teeth [37]. Nevertheless, the model allows controlled assessment of drug diffusion and microbial suppression in a reproducible setting. Future studies using extracted teeth, animal models, or split-tooth designs could help validate the efficacy and distribution of CHX in dentin and pulp-like environments.

Unlike several previously reported intracanal delivery systems that require additional placement steps or specialized activation protocols, the present SRD approach incorporates the drug delivery function directly into a conventional obturation material. By transforming gutta-percha into a localized drug reservoir, the system may reduce technique sensitivity while maintaining prolonged antimicrobial pressure within the canal system. This integration may simplify clinical workflows compared with injectable gels or other delivery platforms that require additional preparation or activation steps.

Taken together, the present findings position sustained-release CHX coating gutta-percha points as part of a broader class of emerging intracanal drug delivery technologies in an experimental context aimed at overcoming the anatomical, biological, and temporal limitations of conventional endodontic disinfection. By combining prolonged antimicrobial activity with anatomical adaptability and procedural simplicity, SRD-based approaches may represent a clinically relevant evolution rather than a replacement of existing chemo-mechanical strategies.

## 5. Conclusions

This study supports the use of sustained-release chlorhexidine technology as a preliminary proof-of-concept approach that could serve as an adjunctive strategy in root canal disinfection. The system is specifically intended to function as a temporary intracanal dressing prior to obturation, rather than as a permanent filling material. Compared to single-dose CHX and placebo, the CHX-coated gutta-percha points maintained prolonged antibacterial and antifungal effects over 14 days, reducing viable biofilm levels of *E. faecalis* and *C. albicans* even in anatomically complex areas. The present findings are limited to antimicrobial efficacy and diffusion behavior in an in vitro model and do not include direct mechanistic evaluation of CHX activity. The sustained presence of CHX within the root canal system may represent a potential formulation advancement, pending further biological and clinical validation in intracanal therapy by addressing the limitations of short-acting irrigants and medicaments. While the model is in vitro, the findings provide a strong rationale for future in vivo studies and suggest that the integration of sustained-release agents into obturation materials may potentially contribute to improved microbial control by preventing reinfection and enhancing microbial control pending further in vivo and clinical validation.

## Data Availability

The original contributions presented in this study are included in the article. Further inquiries can be directed to the corresponding author.
